# Developing data use capacity in the maternal, newborn, child health and nutrition sector in Malawi, Mali, Mozambique and Tanzania: an evolving strategy

**DOI:** 10.7189/jogh.09-010309

**Published:** 2019-06

**Authors:** Talata Sawadogo-Lewis, Emilia Vignola, Tricia Aung, Rebecca Heidkamp

**Affiliations:** Institute for International Programs, Johns Hopkins Bloomberg School of Public Health, Baltimore, Maryland, USA

Alongside the Sustainable Development Goals is a call for a “data revolution” that will “monitor progress, hold governments accountable, and foster sustainable development” [1]. With recent technological advances, the amount and types of data available to governments have increased rapidly. However, there are gaps in data literacy needed to access, analyze and apply these data to policy and program decision making. In 2014, the United Nation’s Independent Expert Advisory Group on a Data Revolution for Sustainable Development called for data-focused capacity building in low-income countries [1].

The National Evaluation Platform (NEP) aims to improve health and nutrition outcomes in women and children by strengthening the capabilities of government institutions to use data to guide Maternal, Newborn, and Child Health and Nutrition (MNCH&N) policies and programs. From 2014-2018, multi-institutional teams in Malawi, Mali, Mozambique and Tanzania – countries that are diverse geographically, linguistically and epidemiologically to increase the generalizability of the tools and lessons generated by the project – each built their own NEP. Participating institutions in each country included those that support MNCH&N through data collection, financing, policy development and/or program implementation. NEP engaged higher-level MNCH&N decision makers as members of NEP High-level Advisory or Steering Committees (HLAC) and technically-focused mid-level staff as members of NEP Technical Working Groups (TWG). A team of faculty from the Institute for International Programs at Johns Hopkins Bloomberg School of Public Health (IIP-JHU) provided tools, training and mentorship to these teams as part of a capacity building strategy that aimed to keep the NEP “country-led and country-owned”. The overall NEP structure is described in more detail in Heidkamp’s 2017 publication [2].

Here we describe the start and evolution of the NEP capacity building strategy and share key learnings from internal and external assessments across the four countries. Our experience can inform efforts by governments and development partners to catalyze a “data revolution” through investments in public sector capacity building.

## DEFINING CAPACITY BUILDING IN THE NEP

**Figure 1:**
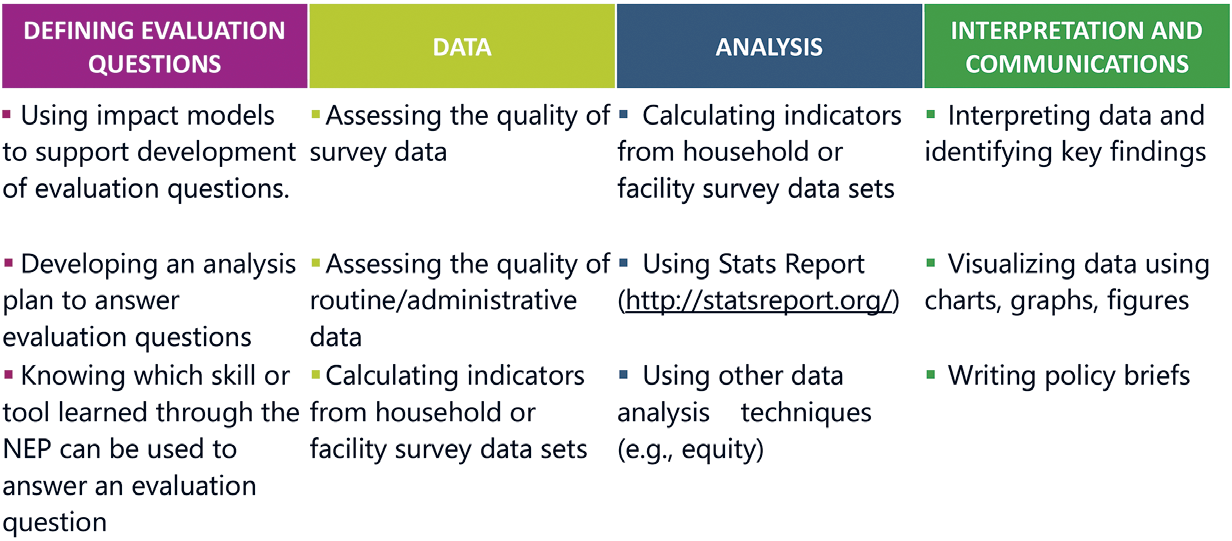
Technical skills by step in National Evaluation Platform (NEP) cycle.

The NEP was ambitious – aiming to develop and capacitate teams drawing members from multiple institutions that would both carry out analyses and effectively engage policy makers with findings. Only at project midterm were we able to clearly articulate the technical (**Figure 1**) and dissemination-oriented skills – by which we mean communication, networking and advocacy skills – required to successfully implement the NEP. Rather, at inception in 2014, we laid out guiding principles for capacity building across the four countries which included:

- Developing multi-institutional teams that could effectively work together and grow less dependent on IIP-JHU technical support across time
- Fostering context-based learning by rooting all capacity building in answering country-specific questions using national data
- Building skills that transfer beyond NEP; all HLAC and TWG members had professional roles that required working with data in some capacity
- Using a “cycle-based” approach to answering questions and progressively introducing knowledge, skills and tools for each step (**Figure 1**): (1) defining priority evaluation questions, (2) accessing and assessing quality of required data; (3) data analysis, (4) communication of key findings
- Adapting country-level capacity building approaches to reflect each team’s questions, priorities and existing skills
- Using a combination of intensive workshops facilitated by IIP-JHU Baltimore and other external partners and day-to-day mentorship channeled through the JHU Resident Advisor in each country

We focused our capacity building efforts on the TWG members rather than on the HLAC. At the individual level, we aimed to improve participant’s core technical skills (**Figure 1**). For institutional capacity building, we aimed for (1) the sustained presence of technical skills within Home Institutions despite turnover in individual TWG members, and (2) a higher-level shift in institutional culture around data for decision making. Our theory of change assumed that building individual capacity would eventually result in improved institutional capacity.

IIP-JHU defined three levels of competency for NEP core technical skills, framed within Bloom’s taxonomy [3]:

- **Level 1:** Remembering and understanding – appreciate concepts and methods. Know who to ask for help and appropriate questions to ask.
- **Level 2:** Applying and analyzing – understand concepts and methods. Ability to do work with guidance.
- **Level 3:** Ability to independently apply skills and complete work.

We expected TWG members to reach level 1 for all skills. We expected Home Institution and some other TWG members to reach Level 2 for most skills. Level 3 is quite advanced and we expected only a few participants to reach it in specific skill areas.

## FINDINGS FROM CAPACITY BUILDING ASSESSMENTS

During the course of the 4-year project we carried out internal and external assessments of the NEP capacity building and refined our strategy based on findings. Internal assessments included country and global workshop evaluations and quarterly IIP-JHU team reflections. We contracted an independent firm, FSG (https://www.fsg.org/) to conduct mid (2014) and end (2017) of grant period evaluations [4,5]. In both rounds FSG visited all four countries and used a combination of individual interviews, focus groups, activity observations, and document review. Interviewees included stakeholders, TWG members, HLAC members, and IIP-JHU faculty members. Despite FSG’s efforts to triangulate information, we recognize that an important limitation to this methodology is that much of the data collected were self-reported information, and are therefore subject to recall bias. The results presented below stem from this external evaluation.

### Mechanisms and evidence for changes in individual and institutional capacity

At endline, FSG used a self-report survey to ask NEP team members how they viewed their own capacity in NEP core skills areas. As hoped, TWG members in the four countries consistently reached level 1. Only a few reached level 2 in select areas, and as expected very few reached level 3 in any area. Given that TWGs function as a unit, as long as members have a complementary set of level 2 skills and are able to access level 3 support, they should be able to complete work. However, at endline, FSG flagged two key issues that threatened sustainability: (1) no country teams achieved complete level 2 skill coverage or had access to level 3 support without IIP-JHU and (2) frequent TWG member turnover was an ongoing challenge.

Most of the focused capacity building for TWG members was delivered through intensive 3-5 day in-person workshops aligned with the NEP cycle. This approach allowed TWG members to be released from their usual work to focus on NEP and for IIP-JHU or other external experts to travel and facilitate trainings. This approach engaged all TWG members in the same way, therefore those who had higher baseline capacity did not necessarily gain new skills. We chose to foster level 1 competency for all rather than level 3 for a few to create a meaningful level of cross-institutional engagement. Turnover created additional challenges as workshop content was cumulative. In some cases, countries responded to these challenges by relying on IIP-JHU mentorship of smaller working groups after the workshops.

At institutional level we did not achieve a sustainable level of technical skills but FSG did find (1) improved collaboration within and across institutions that had not historically worked together and (2) an expanded view of the potential impact of data use.

### Barriers and facilitators to capacity building in the NEP

FSG grounded their endline evaluation in the Learning Transfer Model [6] which broadly assesses factors that lead to successful skill acquisition and use. The model looks at three dimensions, contextualized to NEP:

- **Learning Readiness:** Each individual TWG member’s personal investment with the capacity building efforts (i.e. motivation to learn; intent to use; self-efficacy; career goal alignment).
- **Learning Transfer Design:** The methods and mechanisms used for capacity building (i.e. setting learning goals; practice and modeling; application review).
- **Organizational Alignment:** Organization elements that facilitate and support capacity being built (i.e. manager coaching; peer support; job connection; learning culture)

**Figure:**
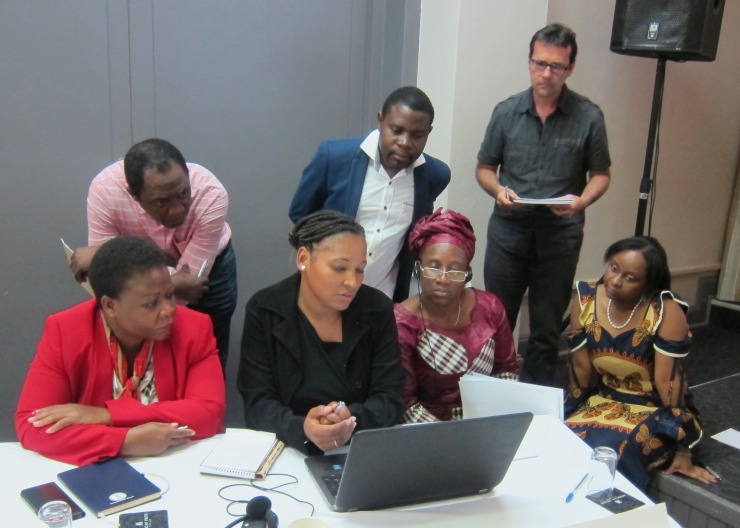
Photo: A Mozambique TWG member explains the work that her team has done on analyzing disease incidence seasonal trends to colleagues from the Malawi and Mali TWGs, IIP-JHU, and external partner at the 2017 NEP Global Collaborators’ meeting (from the collection of Talata Sawadogo-Lewis, used with permission)

In terms of **learning readiness**, motivation to learn was generated in part through accountability to HLACs for answering their questions and the potential for direct impact of findings on their decisions. For most TWG members who work directly with MNCH&N issues, NEP topics readily aligned with their career goals. For those from statistics offices or other institutions more peripheral to MNCH&N, maintaining engagement with the NEP has been more challenging.

As for the **learning transfer design**, IIP-JHU struggled early on with setting learning goals, since at that point NEPs were more theoretical and country priorities had not been defined. In the first cycle of NEP questions, IIP-JHU guided all four countries towards evaluation questions requiring similar tools and training. In subsequent cycles, countries generated evaluation topics with a wider range of methodological approaches and training needs. This slowed the pace of response by the IIP-JHU team as we needed to wait for countries to identify questions and then respond by developing multiple tools and methods concurrently.

Practice and modeling and application review were successfully applied for the skills needed recurrently over the course of the project. Some country teams chose to sub-divide into smaller groups to carry specific tasks forward and thus strengthened related skills. However, this approach came at the expense of the full TWG having this opportunity.

The NEP’s **organizational alignment** varied from country to country, and even within a single country over the course of the project. Turnover in political appointments and institutions’ priorities impacted supervisor and peer support for NEP. Finished products for policy makers – such as policy briefs or finalized reports – were not produced as frequently as hoped due to difficulty in accessing data, complexity of some analyses and/or delays in approvals. Senior-level commitment to the NEP may have waned in between products. To address this, smaller but more frequent products and dissemination events could have been produced to keep the NEP at the forefront of initiatives competing for senior-level attention.

## ADDITIONAL LESSONS LEARNED

We focused capacity building on TWG members assuming that quality TWG outputs would be readily taken up by HLAC. However, not formally including the HLAC level in our capacity building strategy was a missed opportunity. A 2017 study by the NEP Tanzania team on MNCH&N data visualization demonstrated that HLAC-level decision-makers had difficulty interpreting data visualizations commonly used in global health. In response, NEP Tanzania carried out four workshops on interpreting data visualizations during the final months of the project, too late for replication in other NEP countries. A skills assessment of HLAC members earlier in the project might have allowed us to plan more strategically.

This observation extends to TWG members, who also had different capacity levels than originally anticipated. For example, after the first round of country workshops, we recognized that basic Microsoft Excel and Access training was needed across all countries before we could continue. In the future, we would recommend carrying out an initial skills assessment that includes basic data management, analysis and visualization tasks to map baseline level of TWGs.

Furthermore, we would also focus on developing level 2-3 skills in select members who could train and mentor the other TWG members. External experts from IIP-JHU, or preferably national institutions, would still need to provide “level 3” support in select technical areas. This approach is more feasible now that core NEP skills are identified, curriculum has been developed and online collaborative tools such as Stats Report are available [7].

Despite having a wide range of skills within the IIP-JHU team, as the work progressed we identified areas including policy sciences, adult learning, and programming in R where we did not have adequate expertise. We faced challenges training our team members or bringing on consultants to fill the gaps in a timely way. A team attempting a similar endeavor should ensure that expertise gaps are identified and addressed early and assessed throughout the project.

Finally, we had sufficient funding over the course of this grant to allow us the flexibility that this type of project required. We were also purposeful when selecting collaborating institutions, ensuring that the infrastructure to support and interest for our initiative already existed prior to collaboration. We recognize that these are important facilitating factors, and have undoubtedly had an impact on this project’s achievements.

## CONCLUSION

The overarching challenge that we faced in NEP was taking a common vision [8] and applying it to four distinct country-led efforts. Intensive capacity building at the individual and institutional level was crucial to the success and sustainability of the NEP. Moving forward we have a deeper understanding of how to assess and meet capacity needs as well as a better grasp of helpful tools, including methods and curriculum, to more efficiently and effectively shape and implement a capacity building strategy.

## References

[1] Independent Expert Advisory Group on a Data Revolution for Sustainable Development (IEAG). Available: http://www.undatarevolution.org/wp-content/uploads/2014/11/A-World-That-Counts.pdf. 2014. Accessed: 7 March 2018.

[2] Heidkamp R (2017). The National Evaluation Platform for Maternal, Newborn, and Child Health, and Nutrition: From idea to implementation.. J Glob Health.

[3] Bloom BS, Engelhart MD, Furst EJ, Hill WH, Krathwohl DR. Taxonomy of educational objectives: Handbook 1: Cognitive domain. London: Longman Publishing Group; 1984.

[4] FSG. National Evaluation Platform: Findings from the End-of-Grant Evaluation. 2017.

[5] Vignola E, Parkhurst M, Misomali A, Heidkamp R (2018). Encouraging local ownership of an externally-coordinated capacity building initiative in Malawi, Mali, Mozambique, and Tanzania: an exercise in process evaluation.. J Glob Health.

[6] Leimbach M. Learning transfer model: A research-driven approach to enhancing learning effectiveness. Bingley: Emerald Group Publishing Limited; 2010;42:81-6.

[7] Wilson E, Park LA, Zeger S, Roberton T (2017). Reimagining statistical analysis for evidenced-based policy making: Early experiences using Stats Report.. J Glob Health.

[8] Victora CG, Black RE, Boerma JT, Bryce J (2011). Measuring impact in the Millennium Development Goal era and beyond: a new approach to large-scale effectiveness evaluations.. Lancet.

